# The Emblematic Year of 2011, Trials and the 50 Years of Heart Transplantation: Three Relevant Issues

**DOI:** 10.21470/1678-9741-2017-0507

**Published:** 2017

**Authors:** Domingo M. Braile, Paulo Roberto B. Évora

**Affiliations:** 1 Editor-in-Chief - BJCVS; 2 Editor-in-Chief Interim - BJCVS

Writing a scientific editorial is an arduous task, as it should go beyond a summary of the focused journal. Therefore, we have designed this text oriented to three targets: 1) What represents the year of 2011 for the Brazilian Journal of Cardiovascular Surgery (BJCVS); 2) The BJCVS support for feasibility of trials, and; 3) The 50 years of heart transplants.

## The Year of 2011: a Celebration and a Milestone

**Fig. 1:**
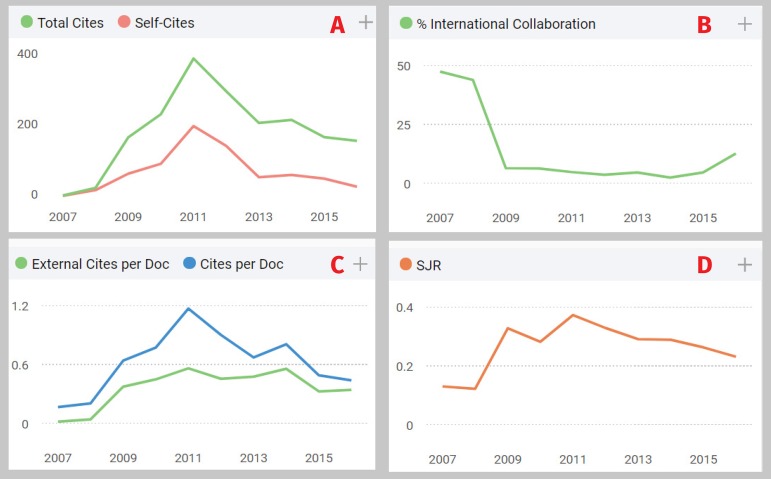
SciELO database: A) Journal self-citation is defined as the number of citations from a journal citing the article to articles published in the same journal; B) International collaboration accounts for the articles that have been produced by researchers from several countries; C) External citations are calculated by subtracting the number of self-citations from the total number of citations received by the journal's documents, and; D) SJR is a measure of scientific influence of journals that accounts for both the number of citations received by a journal and the importance or prestige of the journals where such citations come from.

During the 25-year BJCVS celebrations in 2011, we had the first Impact Factor (IF) published by ISI-Thomson Reuters. The prime number of 0.963, almost 1, at first evaluation, shows the high degree of development of Brazilian cardiovascular surgery. In an attempt to check the SciELO database and verify if it coincides with the 25 years of the BJCVS, we observe the best academic performance of the BJCVS (Figure 1). It should be noted that this performance was independent of international collaboration (Figure 1B). This fact deserves careful evaluation to guide our editorial planning.

## Protocol Trial

Recently, Rodrigues et al.^[1]^ investigated in a Brazilian two-center study the relationship between pre-frailty and adverse postoperative outcomes following cardiovascular surgery. The authors emphasized that this group had more cumulative events than the non-frail group, both in stroke and death rates, and that pre-frail patients had prolonged mechanical ventilation time and hospital length of stay compared with non-frail patients. The authors pointed out that these findings are strongly relevant and contribute to understanding whether the extent of premorbid deficit accumulation adds prognostic value after cardiovascular surgery. In this scenario, BJCVS was chosen by Mejía et al.^[2]^ as a vehicle for the publication of the study protocol of the randomized controlled multicenter FRAGILE trial, whose primary purpose will be to compare adverse cardiac and cerebrovascular events after CABG (on-pump *vs.* off-pump) in pre-frail and frail patients, inaugurating this new type of publication in our medical journal (page 428).

## Fifty Years of Heart Transplantation

This year we celebrate 50 years of the first inter-human heart transplantation. In this edition, Prof. Noedir Stolf reviews the most important steps of this glorious journey. Repeating his words: "Heart transplantation first performed in the course of experiments of other nature at the beginning of 20^th^ century, seen as a speculation for the future in the middle of the same century, is now widely accepted by medical and lay communities as a valuable therapeutic procedure." The heart transplantation is one of the most exciting and remarkable chapters in the history of cardiac surgery.

## Additional Articles in This Issue

This issue of BJCVS presents a selection of various articles that will surely please their readers.

The following articles were chosen for publication: two articles on the use of artificial cardiac stimulation; two articles on surgery in congenital heart diseases; two articles related to aortic diseases; two comparative articles between percutaneous transluminal coronary angioplasty (PTCA) and surgery, and; some general aspects related to cardiac surgery (circulatory support, operations in Jehovah's Witness patients, intensive care unit stay, aspects of heart surgery in the fragile patient).

- The articles on electrical stimulation addressed two issues: one on the evolution to atrial fibrillation with VDD and DDD pacemakers, and the other on the implantation of cardioverter-defibrillators therapy in patients with nonischemic heart failure.
- The articles on congenital heart disease present a casecontrol on the always exciting subject that is the formation of chylothorax as a complication of the surgical correction of congenital heart diseases.
- On diseases of the aorta, we discuss the percutaneous coronary intervention and drug-eluting stents or coronary artery bypass grafting surgery for unprotected left main coronary artery stenosis.
- Impact of myocardial revascularization method on smoking cessation: CABG *versus* percutaneous coronary intervention is another subject under discussion in this issue of BJCVS.

**Domingo M. Braile** ^1^Editor-in-Chief - BJCVS**Paulo Roberto B. Évora** ^2^Editor-in-Chief Interim - BJCVS
